# Re-examining HSPC1 inhibitors

**DOI:** 10.1007/s12192-017-0774-0

**Published:** 2017-03-02

**Authors:** Sheah Lin Lee, Nina Claire Dempsey-Hibbert, Dale Vimalachandran, Terence David Wardle, Paul A. Sutton, John H. H. Williams

**Affiliations:** 1 Chester Centre for Stress Research, Institute of Medicine, University of Chester, Bache Hall, CH2 1BR Chester, UK; 2 University Hospital Southampton, Tremona Road, SO16 6YD Southampton, UK; 3 Centre for Biomedicine Research, Manchester Metropolitan University, Chester Street, M1 5GD Manchester, UK; 4 Countess of Chester Hospital, Liverpool Rd, CH2 1UL Chester, UK

**Keywords:** Heat shock proteins, Colorectal cancer, Chemotherapy, Tumour evolvability, Chemoresistance, Novel target

## Abstract

HSPC1 is a critical protein in cancer development and progression, including colorectal cancer (CRC). However, clinical trial data reporting the effectiveness of HSPC1 inhibitors on several cancer types has not been as successful as predicted. Furthermore, some N-terminal inhibitors appear to be much more successful than others despite similar underlying mechanisms. This study involved the application of three N-terminal HSPC1 inhibitors, 17-DMAG, NVP-AUY922 and NVP-HSP990 on CRC cells. The effects on client protein levels over time were examined. HSPC1 inhibitors were also applied in combination with chemotherapeutic agents commonly used in CRC treatment (5-fluorouracil, oxaliplatin and irinotecan). As HSPA1A and HSPB1 have anti-apoptotic activity, gene-silencing techniques were employed to investigate the significance of these proteins in HSPC1 inhibitor and chemotherapeutic agent resistance. When comparing the action of the three HSPC1 inhibitors, there are distinct differences in the time course of important client protein degradation events. The differences between HSPC1 inhibitors were also reflected in combination treatment—17-DMAG was more effective compared with NVP-AUY922 in potentiating the cytotoxic effects of 5-fluorouracil, oxaliplatin and irinotecan. This study concludes that there are distinct differences between N-terminal HSPC1 inhibitors, despite their common mode of action. Although treatment with each of the inhibitors results in significant induction of the anti-apoptotic proteins HSPA1A and HSPB1, sensitivity to HSPC1 inhibitors is not improved by gene silencing of HSPA1A or HSPB1. HSPC1 inhibitors potentiate the cytotoxic effects of chemotherapeutic agents in CRC, and this approach is readily available to enter clinical trials. From a translational point of view, there may be great variability in sensitivity to the inhibitors between individual patients.

## Introduction

Heat shock proteins (HSPs) are highly conserved molecular chaperones that prevent proteins from misfolding and aggregation. HSPs are classified into several subgroups or “families,” originally named according to their molecular weight but now designated based upon sequence homology (Kampinga et al. 2009). Although first discovered to be upregulated upon elevation of temperature, HSPs have distinct roles in regulating cell functions including proteostasis, apoptosis and carcinogenesis (Hartl et al. 2011; Kennedy et al. 2014; Rappa et al. 2012). Three families of HSPs, HSPC, HSPA and HSPB, receive special attention in cancer due to their implications in diagnosis, prognosis and treatment (Ciocca and Calderwood 2005; Hong et al. 2013).

The HSPC (Hsp90) family is amongst the most abundant proteins in eukaryotic cells with expression being regulated by heat shock factor 1 (HSF1) (Csermely et al. 1998; Lindquist 1986). There are two major isoforms of HSPC in the cytoplasm, which are the stress-inducible HSPC1 (HSP90AA1/Hsp90α) and the constitutive HSPC3 (HSP90AB1/Hsp90β) (Chen et al. 2005). HSPC1 particularly plays a fundamental role in the maintenance of tumour cell characteristics (Tatokoro et al. 2015). Along with other HSPs, it is involved in each of the hallmarks of cancer (Hanahan and Weinberg 2000, 2011) essential for the development of a malignant phenotype.

HSPC1 are found to be elevated in colorectal cancer (CRC) cells compared with corresponding normal cells (Kanazawa et al. 2003; Milicevic et al. 2008; Liu et al. 2010; Wang et al. 2012). A wide variety of oncoproteins are chaperoned by HSPC1, maintaining their stability and function (Citri et al. 2004; Banerji et al. 2008; Azoitei et al. 2012), and in CRC, this is no exception. HSPC1 stabilises mutant proteins in multiple commonly aberrant pathways contributing to carcinogenesis in CRC (Lee et al. 2015). Consequently, the clinical application of HSPC1 inhibitors in the treatment of CRC may prove beneficial.

Many current anti-cancer treatments are targeted towards a single specific defect or oncoprotein. Although this can be successful (Hecht et al. 2009; Flaherty et al. 2010; Kantarjian et al. 2013), the accumulation of oncogenic mutations during cancer progression may render the treatment ineffective due to secondary mutations in the target binding domain and activation of alternative signalling pathways (Shah et al. 2002; Nazarian et al. 2010). Modulating HSPC1 activity, a protein central to many such signalling pathways, has therefore become an attractive method to overcome drug resistance and tumour evolvability. This has led to the development of a number of HSPC1 inhibitors, targeting either the N-terminal or the C-terminal domain (Tatokoro et al. 2015). Two early N-terminal inhibitors in clinical use were the geldanamycin derivatives 17-allylamino-17-demethoxygeldanamycin (17-AAG) and 17-dimethylaminoethylamino-17-demethoxygeldanamycin (17-DMAG). Both of these showed moderate success in clinical trials, both in isolation and in combination with common chemotherapeutic agents (Kim et al. 2009). However, several limitations were evident including hepatotoxicity, solubility issues and reliance on NAD(P)H:quinone oxidoreductase 1 (Kelland et al. 1999; Ge et al. 2006; Solit et al. 2008). Although theoretically an attractive strategy, success rates from clinical trials have not been as predicted. HSPC1 inhibitors show moderate success as monotherapy in lung cancer with ALK re-arrangement and in combination with transtuzumab in HER2+ breast cancer (Modi et al. 2011; Socinski et al. 2013). As a result, attention was shifted to the development of small entity, non-geldanamycin derivates such as NVP-AUY922 and NVP-HSP990 with the aim of improving therapeutic efficacy and reducing toxicity.

Inhibition of HSPC1 function results in degradation of client proteins including HER2, p-Akt, p-S6, HIF1-α, VEGF and a consequent reduction in cell viability (Okui et al. 2011). However, the time course of these degradation events is still unclear. Furthermore, an additional consequence of HSPC1 inhibition is an increase in the anti-apoptotic proteins HSPA1A and HSPB1, via de-stabilisation of the HSF1-HSPC1 complex (Neckers and Workman 2012). Studies that are emerging show that targeting HSPB1 or HSPA1A enhances cytotoxic effects of HSPC1 inhibitors (Powers et al. 2008; Lamoureux et al. 2014).

The aim of this study was to determine whether resistance to chemotherapeutic agents can be overcome by inhibiting the activity of this essential chaperone during a period of such intense cellular stress. In addition, the importance of HSPA1A and HSPB1 in resistance to HSPC1-induced cell death—and resistance to common chemotherapeutic drugs—was investigated. This study involved the application of three HSPC1 inhibitors, 17-DMAG, NVP-AUY922 and NVP-HSP990, to CRC cells. The production of these three inhibitors has been halted for various reasons including failure to show meaningful clinical results and also associated toxicities. Their employment in this study was as a proof of principle to elucidate the effect that different HSPC1 inhibitors, with similar modes of action, have on client proteins that are essential to carcinogenesis. A deeper understanding of HSPC1 inhibitor-induced cellular changes is essential to drive forward research and development of this class of drug with the potential to simultaneously target many signalling pathways shown to be de-regulated in cancer. Changes in client protein levels over time were examined, along with the resultant effects on cell survival, to provide an explanation of why these inhibitors are not as effective in practice as is predicted theoretically. Cell sensitivity to combination treatments involving these HSPC1 inhibitors and chemotherapeutic drugs commonly used in CRC treatment was also analysed.

## Methods

### Cell culture

HT29 cells (ECACC) were maintained at 37 °C in EMEM media (Lonza) supplemented with 10% FBS (Lonza) (10% EMEM) in a humidified environment of 5% CO_2_ in air. Cells were routinely passaged when at 90% confluence using trypsin/EDTA (Lonza).

### Drug treatments

A cell density of 3 × 10^4^ cells/well was seeded into 96-well plates and left to adhere to the plate overnight at 37 °C. 17-Dimethylaminoethylamino-17-demethoxygeldanamycin (17-DMAG) (Bioquote, *SIH-114A*), NVP-AUY922, NVP-HSP990 (both provided by Novartis), oxaliplatin (OX) (Sigma, O9512), 5-fluorouracil (5-FU) (Sigma, F6627), irinotecan (IRN) (Sigma, I1406) and cyclohexamide (CHX) (Sigma, C4859) were all made up to the appropriate concentrations in 10% EMEM and added to the cells for the indicated times. For combination experiments, HT29 cells were treated with 5-FU, OX or IRN for 24 h followed by addition of HSPC1 inhibitors for 48 h. Concentrations of HSPC1 inhibitors (17-DMAG, NVP-HSP990 and NVP-AUY922) and traditional chemotherapeutic agents (5-FU, oxaliplatin and irinotecan) are stated in each figure legend.

### Flow cytometry analysis

Following treatments, cells were trypsinised, transferred to 96-well V-bottom plates and centrifuged at 500*g* for 5 min to remove trypsin/EDTA to be stained with the appropriate antibodies. All analysis was performed on a FACS Canto II flow cytometer (Becton Dickinson).

### Caspase-3, HSPA1A and HSPB1 analyses

A 70 μl/well volume of Cytofix/Cytoperm (BD Biosciences) was added to each well, and the cells were incubated at 4 °C for 20 min. Following further centrifugation, cells were incubated in 100 μl/well of wash buffer (5% FBS in DPBS) for 15 min at room temperature (RT). Cells were centrifuged again before addition of 6.7 μl/well of FITC-conjugated anti-human active caspase-3 (BD Biosciences, 559,341), 50 μl/well of FITC-conjugated anti-human HSPA1A (Bioquote, SMC-103B) or 50 μl/well of FITC-conjugated anti-human HSPB1 (Bioquote, SMC161). The HSP antibodies were diluted 1:50 in wash buffer prior to addition to the wells. Cells were incubated for 45 min at 4 °C in the dark. A 100-μl volume of wash buffer was added to the top of the wells to dilute any unbound antibody before the plate was centrifuged again. Finally, the cells were re-suspended in 100 μl/well of DPBS ready for flow cytometry.

### Phosphorylated NF-ĸB and HER-2 analyses

Cells were fixed using 100-μl/well volume of 4% paraformaldehyde (BD Biosciences), and the cells were incubated at 37 °C for 10 min. Following further centrifugation, cells intended for phosphorylated NF-ĸB analysis were permeabilised using 100-μl/well Perm Buffer III (BD Biosciences, 558,050) and incubated for 30 min at 4 °C. Following permeabilisation (or immediately following paraformaldehyde fixation for HER-2 samples), cells were re-suspended in 100 μl/well of wash buffer for 15 min at RT. The cells centrifuged again, and 4 μl/well of FITC-conjugated anti-human HER-2/NEU (BD Biosciences, 340,553) or 50 μl/well of 1:50 dilution of Alexa-Fluor 488-conjugated anti-human NF-ĸB (BD Biosciences, 558,421) was added. The cells were incubated for 45 min at 4 °C in the dark. A 100-μl volume of wash buffer was added to the top of the wells to dilute any unbound antibody before the plate was centrifuged again. Finally, the cells were re-suspended in 100 μl/well of DPBS ready for flow cytometry.

### Annexin V analysis

Following an additional washing step in DPBS, 50 μl/well of a 1:20 dilution of FITC-conjugated Annexin V (BD Biosciences, 556,419) diluted in binding buffer (0.1 M HEPES/NaOH, 1.4 M NaCl, 25 mM CaCl_2_) was added to each well. The plate was incubated in the dark for 20 min. The cells were analysed immediately by flow cytometry.

### Propidium iodide plate-based assay

Following treatments, the supernatant was removed from each well and 50 μl/well of fresh 10% EMEM was added. A 50-μl volume of a 5 μg/ml propidium iodide solution (Sigma, *P4170*), diluted in DPBS, was added to each well, and the plate was incubated in the dark for 1 h at RT. The fluorescence was detected at Ex/Em 535/617 on a HT Synergy fluorescence plate reader.

### MTS assay

The supernatant was removed from each well following treatments, and 100 μl/well of fresh 10% EMEM was added. A 20-μl volume of a MTS solution (Promega, G1112) was added to each well, and the plate was incubated in the dark for 1 h at 37 °C. The absorbance was detected at 490 nm on a Synergy HT plate reader.

### Western blotting

Western blots were performed on cell extracts from HT29. Protein concentrations of extracts were determined by Uptima CooAssay Max Protein Assay Kit (Interchim, UP87542A) to ensure equal loading. Proteins were separated by electrophoresis on 12.5% sodium dodecyl sulfate polyacrylamide gel electrophoresis (SDS-PAGE) for HSPB1 detection and 10% SDS-PAGE for HSPA1A detection. HSPB1 (StressMarq, SPR-105B) and HSPA1A (StressMarq, SPR-103B) human recombinant proteins were used as standard controls. Separated proteins were transferred onto nitrocellulose membranes using a Bio-Rad Trans-Blot Turbo Transfer System. HSPB1 was detected using a monoclonal HSPB1 antibody (StressMarq, SMC-161A) at 1:5000 dilution as the primary antibody and anti-mouse IgG–peroxidase (Sigma-Aldrich, A5278) at 1:2000 as the secondary antibody. HSPA1A was detected by HSPA1A-DEG-EI (in house) diluted at 1:2000 as the primary antibody and ExtraAvidin®-Peroxidase (Sigma-Aldrich, E2886) diluted 1:5000 as secondary antibody. Membranes were washed with TBS-Tween 20 and then incubated for 5 min in SuperSignal West Femto Maximum Sensitivity (Thermo Scientific) substrate. Chemiluminescence was detected using the ChemiDoc MP detection system (Bio-Rad, Hercules, CA, USA).

### Small interfering RNA transfection

Cells were seeded at a density of 3 × 10^4^ cells/well in 96-well tissue culture plates the day before the experiment. The following day, EMEM was removed from the cells (plate 1) and 100 μl of new EMEM was added to each well. A lipid-based transfection method was used to transfect cells with small interfering RNAs (siRNAs). For each well, 1.4 μl of the transfection reagent, lipofectamine 2000 (Life Technologies, 11,668–027), was diluted in 25 μl of optiMEM (Life Technologies, 31,985), and the solution was applied to a fresh 96-well plate. For each well, a 0.4-μl volume of the respective siRNAs (Dharmacon, M-005168-01-0020 and M-005269-01-0020) was diluted in 25 μl optiMEM and combined with the lipofectamine 2000 solution in the new 96-well plate. This plate was then incubated at RT for 20 min on a plate shaker to allow the lipofectamine and siRNAs to form complexes. After 20 min, the lipofectamine-siRNA solution (50 μl/well) was transferred directly to the corresponding wells containing HT29 cells in the original 96-well plate (plate 1). The cells were then incubated at 37 °C for 24 h before further treatments. In all experiments involving siRNA, control samples treated with (a) transfection agent only, (b) non-targeting siRNA (Dharmacon, D-001210-01-20) and (c) RISC-free siRNA (Dharmacon, D-001220-01-20) were included.

### Statistical analysis

Statistical analyses were performed using PRISM version 5.0. An unpaired *t* test or a one-way ANOVA with a Dunnett’s post hoc test was used as indicated. Statistical significance was considered when *p* < 0.05, and significance levels are indicated on the figures; * represents *p* < 0.05, ** represents *p* < 0.01 and *** represents *p* < 0.001.

## Results

### CRC cells show differential sensitivity to HSPC1 inhibitors

**Fig. 1:**
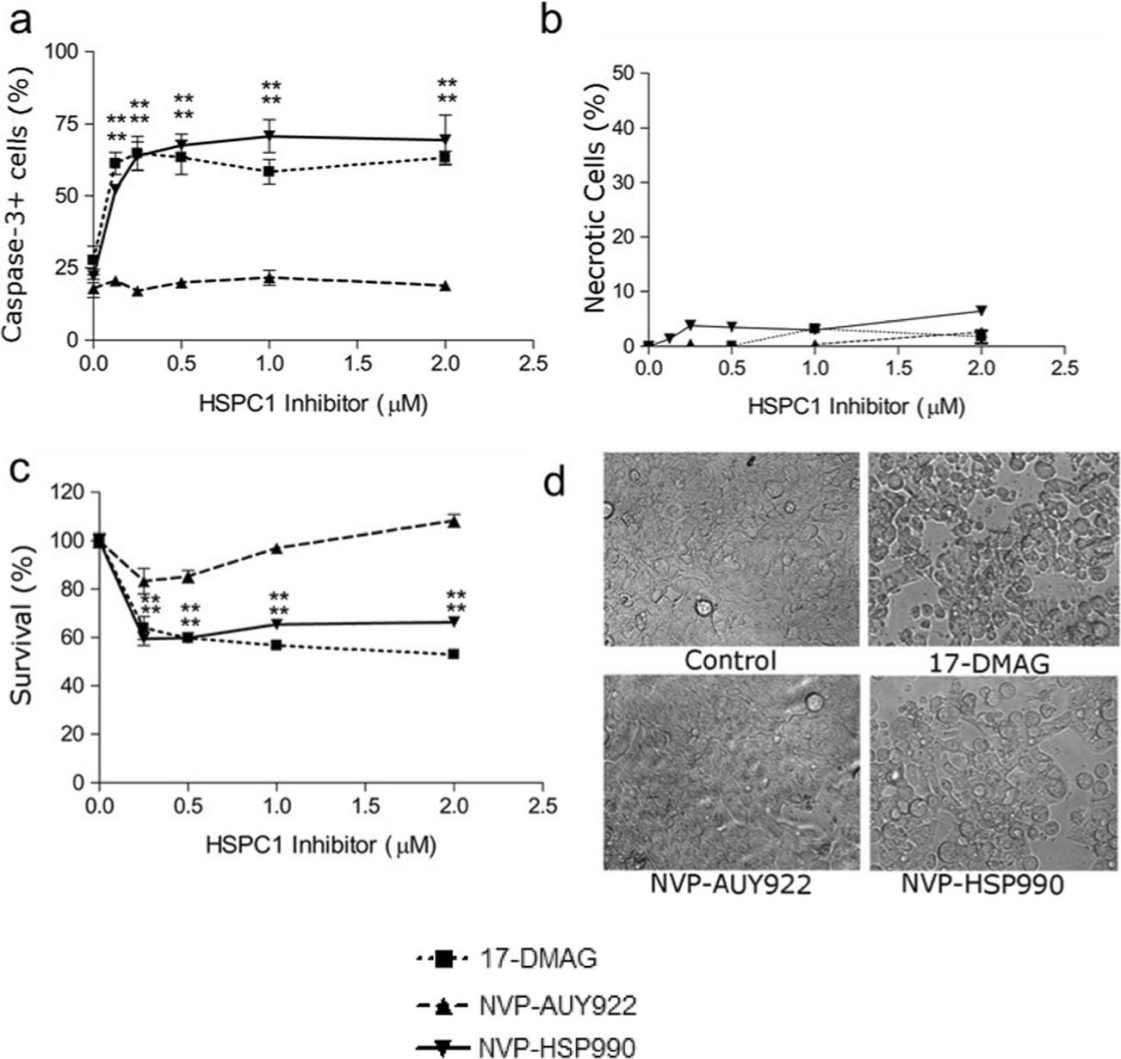
**a** Caspase-3. **b** Propidium iodide. **c** MTS assays on HT29 cells treated with HSPC1 inhibitors (0–2 μM) for 48 h. **d** Bright field microscopy at ×40 magnification on HT29 cell treated with HSPC1 inhibitors (1 μM) for 48 h. Data in **a**, **b** and **c** are presented as mean ± SEM. Statistical analysis was performed using the one-way ANOVA with Dunnett’s post hoc test

HT29 cells were treated with varying concentrations of the HSPC1 inhibitors 17-DMAG, NVP-AUY922 and NVP-HSP990 for 48 h. Apoptosis, necrosis and cellular biochemical activity were assessed using the caspase-3, PI and MTS assays, respectively. A significant increase in caspase-3 levels was observed following 125-nM treatment with 17-DMAG and NVP-HSP990, whilst NVP-AUY922 had no effect even at 2 μM concentrations (Fig. 1a). Significant PI uptake was not observed (Fig. 1b) following any of the inhibitors. MTS assay showed a significant decrease in biochemical activity and hence cell survival following treatment with nanomolar concentrations of 17-DMAG and NVP-HSP990 but not NVP-AUY922 (Fig. 1c). Bright field microscopy of the cells highlighted the drug resistance displayed by HT29 cells to NVP-AUY922 and their sensitivity to 17-DMAG and NVP-HSP990 (Fig. 1d). Taken together, the results indicate that 17-DMAG and NVP-HSP990 induce cytostasis and cell death in HT29 cells via apoptosis and not via necrosis. HT29 cells appear to be resistant to NVP-AUY922.

### HSPC1 inhibitors act to de-stabilise HSPC1 client proteins at an early stage of treatment

**Fig. 2:**
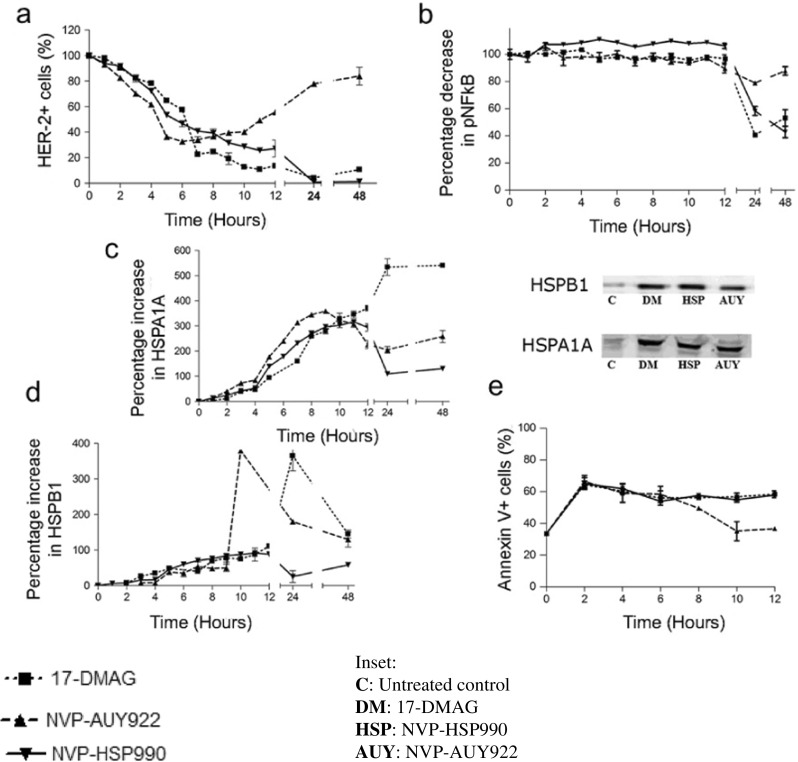
Time course analysis of **a** HER-2, **b** pNF-kB, **c** HSPA1A, **d** HSPB1 and **e** Annexin V, in HT29 cells treated with HSPC1 inhibitors (1 μM) for 48 h. Data are presented as mean ± SEM. Statistical analysis was performed using the one-way ANOVA with Dunnett’s post hoc test. The *inset* denotes the Western blots for HSPB1 and HSPA1A following 48 h of HSPC1 inhibitor treatment (1 μM)

Levels of HER-2 and pNF-ĸB (p65 subunit) were analysed in HT29 cells over the course of treatment with 1-μM HSPC1 inhibitor. HER-2 levels were significantly reduced following just 2 and 3 h of treatment with 17-DMAG and NVP-HSP990, respectively, and continued to decrease throughout the course of 48-h treatment (Fig. 2a). Despite the drug resistance observed earlier, levels of this HSPC1 client protein were also significantly reduced in NVP-AUY922-treated cells during early-stage treatment. However, following 6-h treatment with NVP-AUY922, HER-2 levels began to increase and returned to pre-treatment values at 48 h (Fig. 2a). In comparison, the level of pNF-ĸB, a protein indirectly regulated by HSPC1 via its interaction with IĸK, was unaffected by any of the inhibitors for the first 12 h of treatment (Fig. 2b). Levels did decrease however following 24-h treatment with 17-DMAG, NVP-HSP990 and to a lesser degree NVP-AUY922.

As predicted, due to de-stabilisation of the HSF1-HSPC1 complex, levels of HSPA1A (Fig. 2c) and HSPB1 (Fig. 2d) were significantly increased following 3-h treatment with all three inhibitors, including NVP-AUY922. 17-DMAG was the most effective at sustaining an increase in HSPA1A levels, maintaining a fivefold increase above baseline at 48 h. NVP-HSP990 and NVP-AUY922 were also able to stimulate a threefold increase in HSPA1A within 12 h, which decreased after 24 h. 17-DMAG had stimulated a fourfold increase in HSPB1 above baseline levels at 24 h, which was reversed at 48 h. NVP-AUY922 treatment also resulted in a sudden fourfold increase at 10 h followed by a decline similar to that observed after 17-DMAG treatment. In contrast, NVP-HSP990 treatment resulted in a much more gradual increase in HSPB1 which eventually decreased after 24 h. These changes in HSPB1 levels highlight the differences in cellular response induced by each inhibitor, despite the similar modes of action, as mentioned above. Flow cytometry data showed that HSPA1A was induced to a much greater extent than HSPB1 following HSPC1 inhibitor treatment. The stimulating effects of these inhibitors on HSPB1 and HSPA1A were further confirmed using western blotting, although differences between the two proteins were not as prominent when analysed using this less sensitive technique (Fig. 2, inset).

Examining the early apoptotic state of HT29 cells during inhibitor treatment (Fig. 2e), using Annexin V time-course analysis, showed that although phosphatidylserine (PS) levels increased significantly following all three inhibitors, it began to decrease after 6 h of NVP-AUY922 treatment. This time point coincided with the time point when NVP-AUY922 started to lose its HER-2 degradation ability (Fig. 2a), suggesting that the poor cytostatic and cytotoxic effects of NVP-AUY922 might be related to its inability to degrade client proteins.

### The increase in HSPA1A and HSPB1 in response to HSPC1 inhibition is due to protein synthesis as opposed to re-localisation of existing protein

**Fig. 3:**
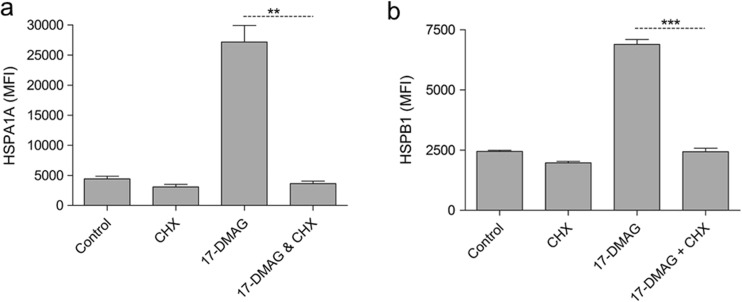
**a** HSPA1A and **b** HSPB1 analyses in HT29 cells treated with CHX (2.5 μg/ml) for 1 h before addition of 17-DMAG (1 μM) for 24 h. CHX remained on the cells throughout the 17-DMAG treatment period. Data are presented as absolute mean fluorescence intensity (MFI) values. Statistical analysis was performed using the unpaired *t* test

HT29 cells were treated with CHX, an inhibitor of protein translation prior to 17-DMAG treatment. As expected, levels of HSPA1A (Fig. 3a) and HSPB1 (Fig. 3b) were significantly increased following 17-DMAG-only treatment whilst prior treatment with CHX prevented the induction of these proteins, confirming that HSPA1A and HSPB1 induction is not due to re-localisation of existing proteins to the cytoplasm.

### HSPC1 inhibitors potentiated the effect of chemotherapeutic agents

The potential for sensitising CRC cells to the chemotherapeutic agents 5-FU, OX and IRN using HSPC1 inhibitors was tested. Dose-response experiments (data not shown) determined the concentrations of chemotherapeutic agents to be used. Non-toxic concentrations were used so that any sensitisation could be successfully observed.

**Fig. 4:**
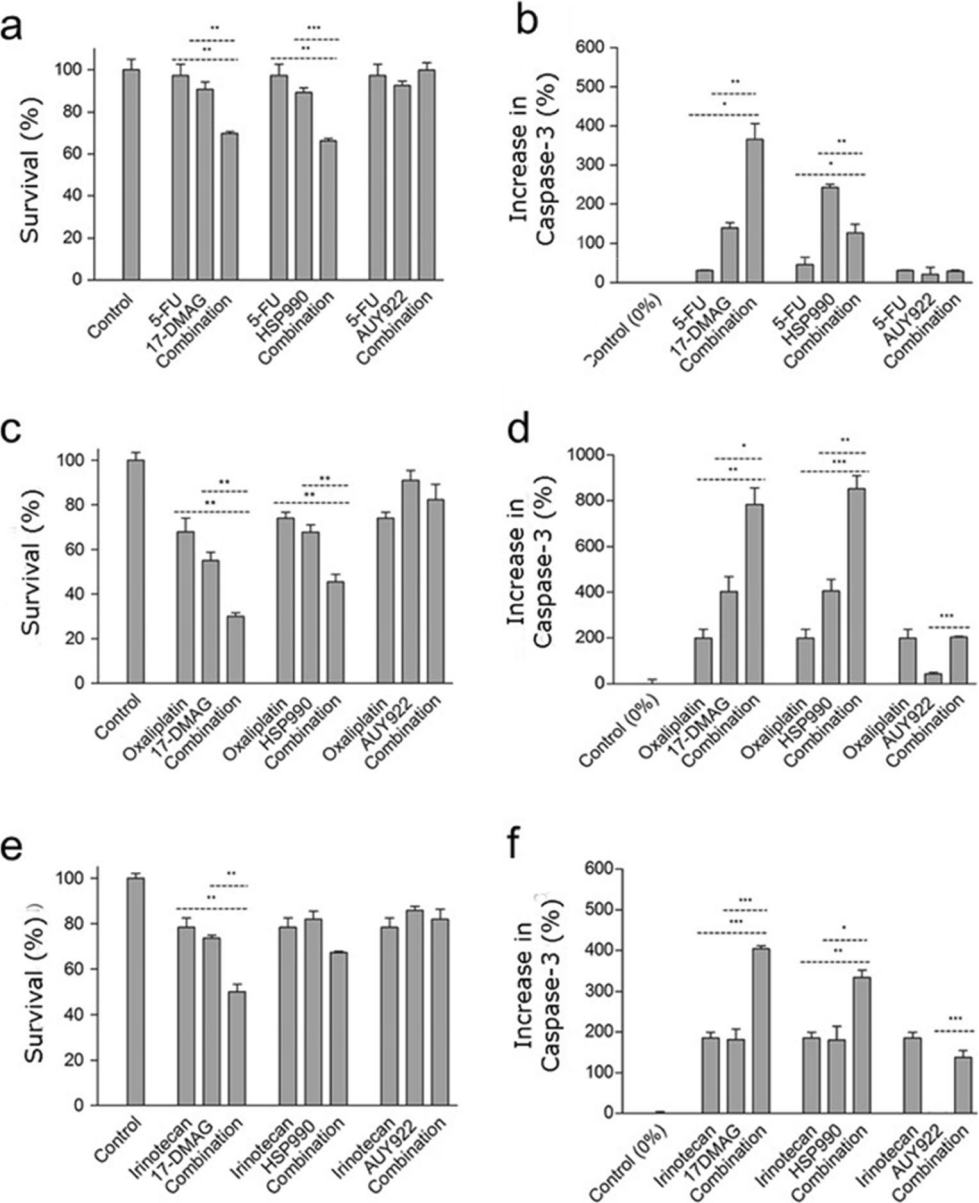
HT29 cellular metabolism (**a**, **c**, **e**) and caspase-3 analysis (**b**, **d**, **f**) following treatment with 12.5 μM 5-FU (**a**, **b**), OX (**c**, **d**) or IRN (**e**, **f**) for 24 h followed by different HSPC1 inhibitors (300 nM) for a further 48 h. Data are presented as percentage of cell metabolism and percentage increase in caspase-3 compared to the control (untreated) population. Statistical analysis was performed using the unpaired *t* test

When combined with 5-FU, 17-DMAG and NVP-HSP990 were able to significantly decrease cell metabolism by a further 20% compared to single agent treatment (Fig. 4a). In contrast, no sensitisation to 5-FU was observed following combined treatment with NVP-AUY922. When looking at apoptosis in these cells, 17-DMAG was able to further increase 5-FU-induced apoptosis significantly, whilst NVP-AUY922 did not potentiate the cytotoxic effect of 5-FU (Fig. 4b). Caspase-3 levels appeared to be lower in combination treatments involving NVP-HSP990 than either agent alone, but this is presumably due to induction of late-stage apoptosis when concentrations of these effector caspases begin to reduce (Fig. 4b). 17-DMAG and NVP-HSP990 were also able to potentiate the cytostatic effect of OX (Fig. 4c) and increased the degree of apoptosis by approximately twofold when compared with OX in isolation (Fig. 4d). Again, NVP-AUY922 was unable to replicate this same potentiation. Sensitisation effects of these inhibitors on IRN were not as pronounced as with 5-FU and OX. Only 17-DMAG showed a significant effect on potentiating the effects of IRN when biochemical activity was analysed (Fig. 4e), yet when caspase-3 levels were analysed, both 17-DMAG and NVP-HSP990 were able to sensitise cells to IRN-induced apoptosis (Fig. 4f). This suggests that the effects are more cytotoxic than cytostatic. Again, NVP-AUY922 did not have any effect on either the cytostatic or cytotoxic effects of irinotecan.

### Sensitivity to HSPC1 inhibitors is not heavily reliant upon HSPA1A or HSPB1 levels

**Fig. 5:**
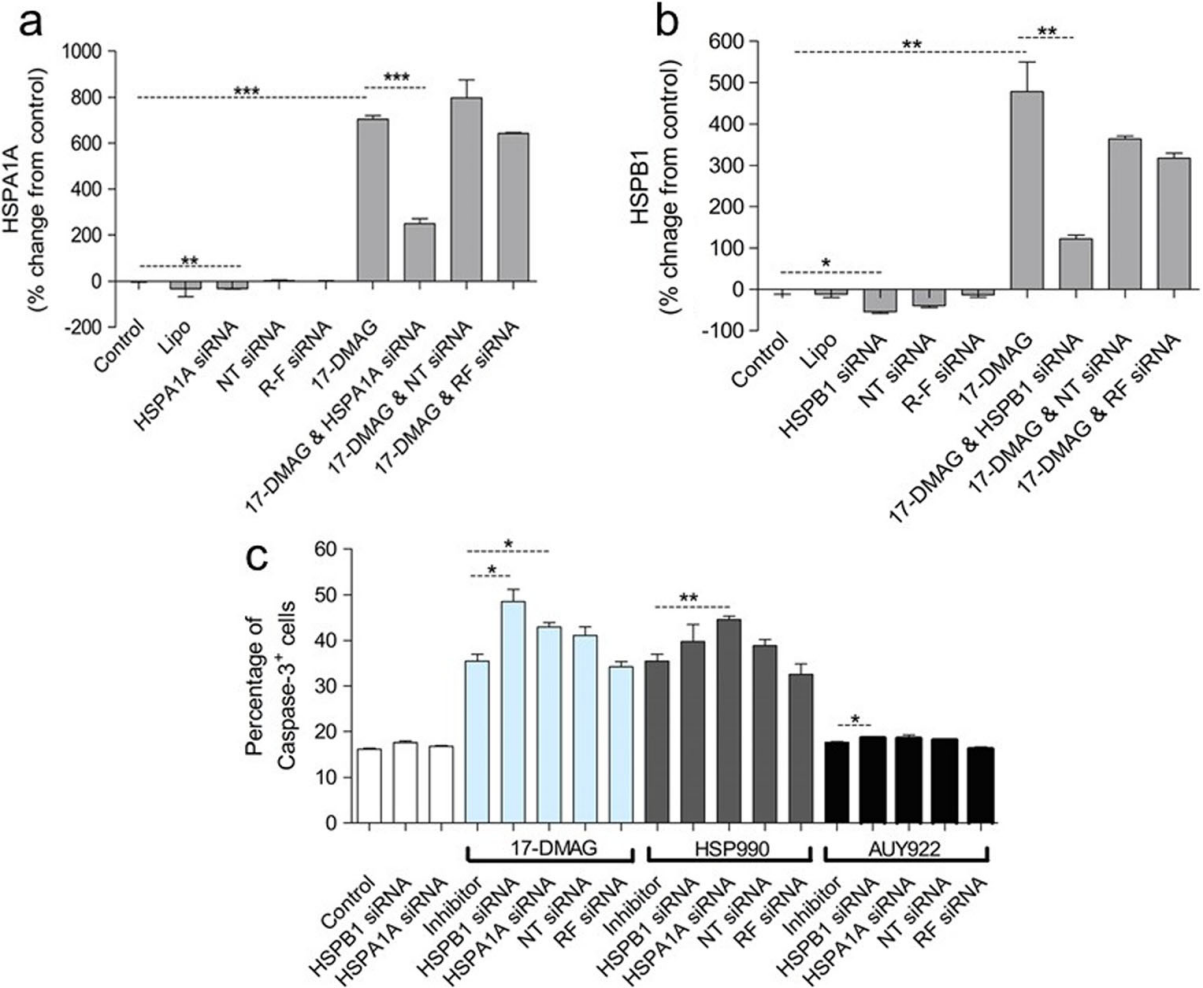
**a** HSPA1A, **b** HSPB1 and **c** caspase-3 analyses in HT29 cells following siRNA-mediated gene silencing prior to HSPC1 inhibitor treatment. Cells were treated with siRNA for 24 h before addition of HSPC1 inhibitor (300 nM) to the culture medium for a further 48 h. Lipofectamine only (Lipo), non-targeting siRNA (NT) and RISC-free siRNA (RF) were used as controls to ensure specific knockdown of HSPA1A or HSPB1. HSPA1A and HSPB1 data are presented as percentage change from levels expressed by untreated cells. Caspase-3 data is presented as percentage of cells that are positive for caspase-3 after 48 h. Statistical analysis was performed using the unpaired *t* test to compare inhibitor-only treatment with the corresponding inhibitor plus siRNA

The increase in anti-apoptotic HSPA1A and HSPB1 levels in response to HSPC1 inhibitor treatment could be counter-productive, hindering the cytotoxic effects of HSPC1 inhibitors. Therefore, *HSPA1A* and *HSPB1* genes were silenced using lipid-based siRNA transfection techniques prior to treatment with HSPC1 inhibitors, and protein levels along with caspase-3 levels were analysed. 17-DMAG was used as a representative HSPC1 inhibitor and, as expected, resulted in a significant induction of both HSPA1A and HSPB1 at the concentration of 300 nM used in the previous set of experiments (Fig. 5a, b). Gene silencing of *HSPA1A* (Fig. 5a) and *HSPB1* (Fig. 5b) showed a significant decrease in protein levels from baseline levels, whilst control siRNAs did not affect these proteins, indicating effective and specific knockdown. When cells were treated with 17-DMAG following siRNA-mediated knockdown of *HSPA1A* (Fig. 5a), there was greater than a fourfold reduction in HSPA1A protein when compared to protein levels in cells treated with 17-DMAG in isolation. Similarly, knockdown of *HSPB1* prior to treatment with 17-DMAG reduced HSPB1 protein levels by fivefold, when compared to levels observed in 17-DMAG-only-treated cells (Fig. 5b). Treating cells with 17-DMAG following transfection with control siRNAs did not affect the levels of HSPA1A or HSPB1 protein when compared to 17-DMAG-only treatment, again, highlighting the specific nature of the gene silencing.

Caspase-3 analysis in these cells revealed that silencing of *HSPA1A* prior to 17-DMAG treatment increased the level of apoptosis from 35% in 17-DMAG-only-treated cells to 50% (Fig. 5c), whilst similar knockdown of *HSPB1* also caused a small but significant sensitisation to 17-DMAG treatment. As expected, caspase-3 levels were not significantly different in cells transfected with the control siRNAs prior to 17-DMAG treatment when compared with 17-DMAG treatment alone, suggesting that it is the specific knockdown of *HSPA1A* or *HSPB1* causing the increased drug sensitivity rather than merely the process of siRNA transfection. However, given the substantial decrease in the anti-apoptotic HSPA1A or HSPB1 proteins using this technique, observing such a small increase in drug sensitivity is somewhat unexpected. Similar experiments using NVP-HSP990 demonstrated that knockdown of *HSPA1A* prior to NVP-HSP990 treatment causes a small but significant increase in caspase-3 levels compared to those levels seen in NVP-HSP990-only-treated cells. In contrast, silencing of *HSPB1* did not sensitise cells to NVP-HSP990 (Fig. 5c). Although silencing of *HSPB1* but not *HSPA1A* prior to NVP-AUY922 treatment resulted in a small increase in caspase-3 levels when compared to NVP-AUY922 treatment alone, HT29 cells continued to show resistance to this HSPC1 inhibitor, with only a 2% increase in caspase-3 levels above control following the double treatments (Fig. 5c).

### Gene silencing of HSPB1, but not HSPA1A, potentiated the effects of 5-FU and OX

To determine whether HSPB1 or HSPA1A are important in determining sensitivity to mainstream chemotherapeutic agents used in CRC treatment, siRNA transfection techniques were used as before to knockdown prior to treatment with 5-FU, OX and IRN.

**Fig. 6:**
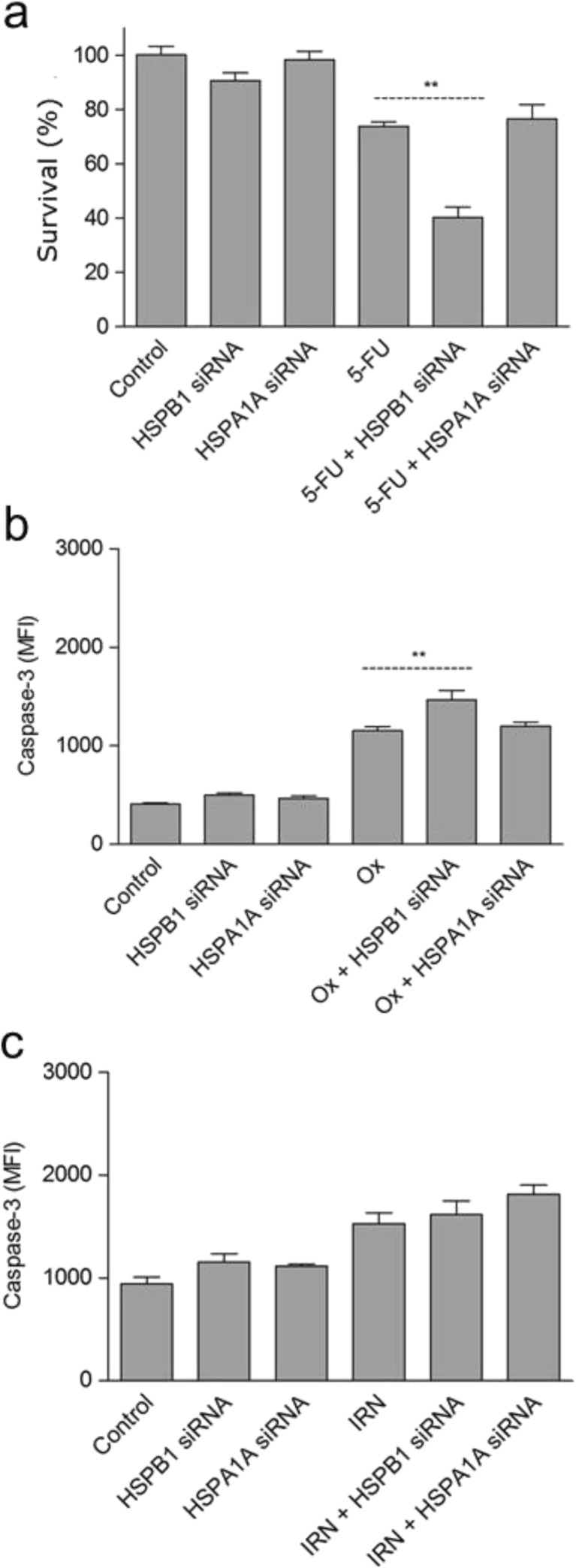
Cell survival analysis analysed by caspase-3 or MTS assay in HT29 treated with HSPB1 or HSPA1A siRNA for 24 h followed by 12.5 μM of **a** 5-FU, **b** OX or **c** IRN for a further 48 h. Statistical analysis was performed using the unpaired *t* test to compare chemotherapeutic drug alone with the corresponding drug + siRNA-treated samples

Cell survival decreased by approximately 30% after 5-FU treatment of HSPB1-knockdown HT29 cells, when compared with 5-FU treatment alone (Fig. 6a). There was, however, no difference in metabolism when *HSPA1A* gene was silenced instead (Fig. 6a), suggesting that HSPB1 may be more important in conferring drug resistance to 5-FU. A similar picture was observed with OX treatment, as caspase-3 levels were significantly higher following OX treatment of HSPB1 knockdown, but not HSPA1A knockdown HT29 cells versus OX-treated cells only (Fig. 6b). Conversely, the cytotoxic effects of IRN were not affected by prior silencing of either HSPB1 or HSPA1A (Fig. 6c).

### Serial re-application of NVP-AUY922 results in increased HSPC1 client protein degradation and cell sensitivity to chemotherapy

**Fig. 7:**
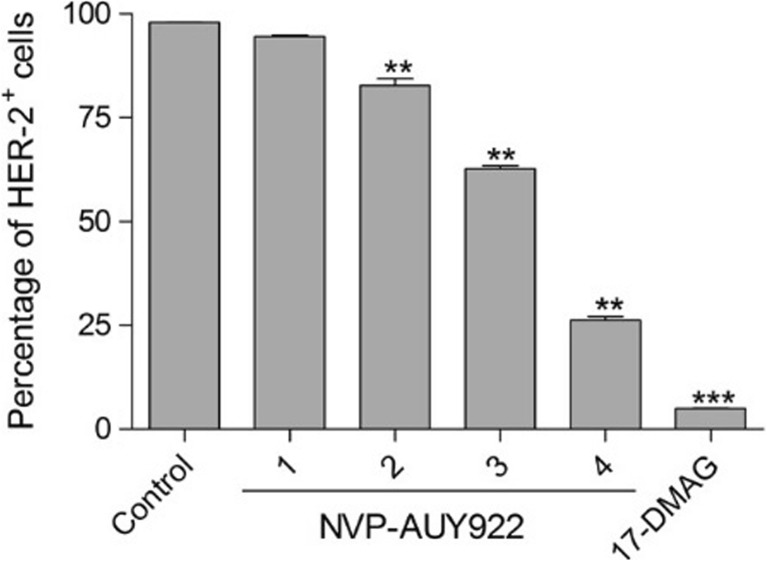
HER-2 levels in HT29 cells treated for 24 h with 1 μM NVP-AUY922 or for comparison, 17-DMAG. NVP-AUY922 was re-applied at varying time intervals during the 24-h period. Twenty-four-hour treatment with NVP-AUY922, no re-application (*1*). NVP-AUY922 was re-applied at *t* = 6 and left for the remaining 18 h (*2*). NVP-AUY922 was re-applied at *t* = 6 and *t* = 12 and left for the remaining 12 h (*3*). NVP-AUY922 was re-applied at *t* = 6, *t* = 12 and *t* = 18 and left for the remaining 6 h (*4*). Statistical analysis was performed using unpaired *t* test to compare percentage changes in HER-2 after various treatment to control (untreated) population

**Fig. 8:**
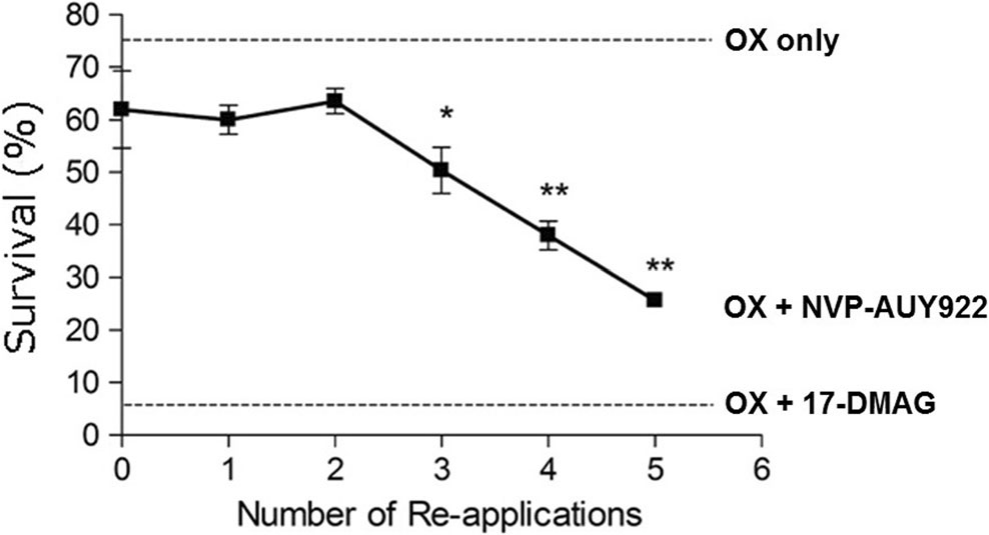
MTS assay on HT29 cells following combined treatment with OX (25 μM) and NVP-AUY922 (300 nM) and for comparison, 17-DMAG (300 nM). OX was applied in isolation for the first 24 h, and NVP-AUY922/17-DMAG was added to the culture media for a further 48 h. NVP-AUY922 was re-applied at specific time intervals over the 48-h period. Forty-eight-hour treatment with NVP-AUY922, no re-application (*0*). NVP-AUY922 was re-applied at *t* = 6 and left for the remaining 42 h (*1*). NVP-AUY922 was re-applied at *t* = 6 and *t* = 12 and left for the remaining 36 h (*2*). NVP-AUY922 was re-applied at *t* = 6, *t* = 12 and *t* = 24 and left for the remaining 24 h (*3*). NVP-AUY922 was re-applied at *t* = 6, *t* = 12, *t* = 24 and *t* = 32 and left for the remaining 16 h (*4*). NVP-AUY922 was re-applied at *t* = 6, *t* = 12, *t* = 24, *t* = 32 and *t* = 40 and left for the remaining 8 h (*5*). Statistical analysis was performed using the one-way ANOVA with Dunnett’s post hoc test, and significant differences from the “OX only” are indicated

So far, HT29 cells have shown resistance to NVP-AUY922, both when applied in isolation and when applied in combination with chemotherapeutic drugs. The effects of this HSPC1 inhibitor on client protein levels appear to be transient. Therefore, the effect of re-applying NVP-AUY922 every 6 h throughout the entire treatment duration was investigated. Interestingly, increasing the number of re-applications over the 24-h period significantly reduced the levels of HER-2 (Fig. 7a). Furthermore, when cells were treated with a combination of OX and NVP-AUY922, where NVP-AUY922 was re-applied at regular time intervals, increasing the number of re-applications resulted in a significant decrease in cell survival with only 30% of cells surviving five re-applications (Fig. 8). However, the cytostatic effect is still inferior when compared to OX in combination with a single dose of 17-DMAG (Fig. 8).

## Discussion

### HSPC1 inhibitors elicit different responses in the same CRC cell line

HSPC1 inhibition in anti-cancer therapy has been well studied, and a number of clinical trials have been carried out using both N-terminal and C-terminal inhibitors on a variety of cancer cell types (Hong et al. 2013). Yet, most trials report a modest response at best to these treatments, and despite very similar mechanisms of action between some inhibitors, there are differences in response rates. The time course of events involving inhibitor interaction with the ATP-binding pocket of HSPC1 and consequent release and degradation of the HSPC1 client (Blagg and Kerr 2006) is unclear and will undoubtedly differ between cancer cell types and between inhibitors. Different responses to HSPC1 inhibitors have been reported in the literature but little attention has been paid to this observation, and its clinical relevance has not been explored. The current study have showed that in the same cell line (HT29), it is possible to be simultaneously susceptible and resistance to the same class of N-terminal HSPC1 inhibitor with similar underlying mechanism.

This differential sensitivity to N-terminal inhibitors has been reported in other studies, although they reported resistance to geldanamycin derivatives and concomitant sensitivity to NVP-AUY922 (Gaspar et al. 2009; Mayor-López et al. 2014; Kim et al. 2015). Niewidok et al. (2012) used the same concentration of NVP-AUY922 and NVP-BEP800, also an HSPC1 inhibitor, and showed distinct difference in plating efficacy as well as Akt and Raf-1 degradation in lung and glioblastoma cell lines. Thus far, clinical trials are designed to use only one type of HSPC1 inhibitor. However, it is possible based on the findings in this study that patients who are resistant to one HSPC1 inhibitor could respond to another. This does raise the next question that needs to be addressed, what is the mechanism of resistance to HSPC1 inhibitors?

#### Resistance to NVP-AUY922 is likely associated with an inability to sustain HSPC1 client protein degradation

The results from the present study suggest that the cytotoxic and cytostatic effects of 17-DMAG and NVP-HSP990 are directly associated with their ability to maintain decreased levels of HSPC1 client proteins. Initial decreases in HER2 and NF-ĸB and concomitant increases in HSPA1A and HSPB1 were observed after treatment with all three HSPC1 inhibitors. However, a striking difference in the time course of client-degradation events following NVP-AUY922 treatment compared to that of 17-DMAG and NVP-HSP990 can be observed after 6–8 h. In particular, the decrease in HER-2 following NVP-AUY922 is seen to be transient, with the level of HER-2 returning to baseline level after 48 h of treatment. These results confirm that the function of HSPC1 inhibitor is mediated through its ability to degrade client proteins and support the findings published in the literature (McLaughlin et al. 2006; Okui et al. 2011; Lee et al. 2011).

Increasing the concentration of NVP-AUY922 applied to the HT29 cells did not overcome this resistance (Fig. 1), yet repeated application of NVP-AUY922 on HT29 cells over a 24-h period resulted in a sustained decrease in client protein levels and a sensitisation to low-dose OX (Fig. 8), an effect that has not been previously investigated. It could be hypothesised that the NVP-AUY922’s affinity for HSPC1 is lower than that of 17-DMAG and NVP-HSP990 in CRC cells, and as a result, it is unable to effectively compete with HSPC1 clients for the HSPC1 ATP-binding site. In agreement with these findings, NVP-HSP990 has been found to be more effective at inducing HSPA1A expression in neuroendocrine carcinoid cells than NVP-AUY922 treated with the same doses, although IC50 values were higher for NVP-HSP990 (Zitzmann et al. 2013). The time course of events was monitored up to 144 h in that study, so extending the treatment duration in the present system may have allowed sufficient time to see NVP-AUY922-associated toxicity. In a study examining the NVP-AUY922-induced degradation of HSPC1 clients in glioblastoma cell lines, effects on some client proteins were seen to be more transient compared to 17-AAG treatment, supporting the results shown here (Gaspar et al. 2010).

#### 17-DMAG and NVP-HSP990 potentiated the effects of 5-FU, OX and IRN

By targeting HSPC1 during conventional chemotherapeutic treatment, it is proposed that normal cell signalling is disrupted, and hence, cells show increased susceptibility to the chemotherapeutic agent. By treating with chemotherapeutic agents first, cells become stressed and ultimately more reliant on HSPs for survival. Subsequent treatment with a HSPC1 inhibitor, in theory, should enhance the cytotoxic effects of the drug. In the present study, both 17-DMAG and NVP-HSP990 potentiated the effects of low-dose 5-FU and OX, and 17-DMAG was also found to enhance the effects of low-dose IRN. The findings are similar to those reported by He et al. (2014), who showed potentiation of capecitabine activity, a 5-FU pro-drug, using the HSPC1 inhibitor ganetespib in HCT116 CRC cells. Additive effects of 17-AAG and SN-38, the active metabolite of IRN, have also been reported in p53-null HCT116 cells (Tse et al. 2009). NVP-HSP990 has also shown synergy when used in combination with melphalan in multiple myeloma cells, inducing significantly more apoptosis than that induced by either agent alone (Lamottke et al. 2012).

In line with the observed resistance following single application of NVP-AUY922, this inhibitor was unable to enhance the cytotoxicity of 5-FU, OX or IRN in HT29. This is explained by the ability of the HSPC1 client proteins to return to baseline levels during single-dose treatment, and therefore, normal cell signalling is restored. However, NVP-AUY922 was able to show activity in other cell lines when combined with chemotherapeutic agents such as temsirolimus in oral squamous cell carcinoma and cytarabine in acute myeloid leukaemia (Okui et al. 2013; Wendel et al. 2016). Therefore, the application of NVP-AUY922 in CRC should be further investigated in other cell lines or xenograft models which may show positive results.

#### HSPA1A and HSPB1 play a role in chemosensitivity to HSPC1 inhibitors and mainstream CRC agents

A hallmark of HSPC1 inhibition is the degradation of the HSPC1-HSF1 complex and resultant HSPA1A (and HSPB1) expression which may attenuate inhibitor treatment (Davenport et al. 2010). This study shows that siRNA-mediated silencing of these genes prior to HSPC1 inhibitor treatment was successful at suppressing the induction of HSPA1A and HSPB1. However, despite highly significant decreases in protein levels, the effect on HSPC1-inhibitor-induced apoptosis was only modest, yet statistically significant, suggesting that induction of these proteins following inhibitor treatment may not be as hampering as predicted. However, only a single gene was silenced in any given sample. Silencing of HSPB1, for example, may result in a compensatory effect by HSPA1A or other HSPs. Future dual-silencing techniques prior to inhibitor treatment may reveal a greater role for these proteins in cytoprotection. The results support findings by Powers et al. (2008), who reported that silencing of HSPA1A did not sensitise HCT116 cells to 17-AAG, yet dual silencing of HSPA1A and HSPA8 (Hsc70) showed significant potentiation.

HSPA1A and HSPB1 have both been implicated in chemoresistance towards 5-FU in CRC (Grivicich et al. 2007; Tsuruta et al. 2008; Ang et al. 2010), whilst Choi et al. (2007) observed that in IRN-resistant CRC cell lines, significantly higher protein levels of HSPB1 were expressed. In the present study, both HSPA1A and HSPB1 were shown to be highly induced following treatment with 5-FU, OX and IRN, which could be prevented by prior siRNA-mediated silencing. This knockdown of HSPB1 was seen to sensitise cells to both 5-FU and OX, yet sensitivity to IRN was not affected. Silencing HSPA1A, on the other hand, did not sensitise cells to 5-FU, OX or IRN. The results suggest that HSPB1 is more important in the present system at conferring resistance to these drugs, and are supported by findings from Sharma et al. (2009) who show increased sensitivity to 5-FU following siRNA silencing of HSPB1, and also DNAJB1 (Hsp40) in hepatoma cells. OGX-427 is an anti-sense oligonucleotide which targets HSPB1. Several clinical trials on OGX-427 for solid tumours have provided positive results (OncoGenex 2016). The results reported in this study provide future justification to examine the efficacy of this drug in CRC.

#### HSPC1 inhibition in future CRC therapy

Despite the excitement surrounding HSPC1 inhibitors as means of overcoming chemoresistance and targeting tumour evolvability, resistance to these inhibitors has been observed. Acquired resistance to geldanamycin derivates has been proposed by some investigators to be due to a decrease in levels of NAD(P)H:quinone oxidoreductase 1 (NQO1) (Kelland et al. 1999; Guo et al. 2005; Gaspar et al. 2009). NVP-AUY922 is thought to be more useful in these cases as it is not reliant on NQO1 for its toxic effects. A study into HSPC1 inhibitor sensitivity in lung cancer cells with ALK re-arrangement showed that NQO1 expression was not associated with acquired resistance to 17-DMAG, and rather, it is the induction of the multidrug resistant protein, P-glycoprotein that confers resistance in these cells (Kim et al. 2015). These findings highlight the different mechanistic actions of these drugs despite similar HSPC1 N-terminal inhibition and suggest that further mechanisms may be discovered that will explain resistance to NVP-AUY922.

Client protein activation by HSPC1 involves a plethora of co-chaperones including Aha1. Binding of Aha1 to HSCP1 is essential as an activator of HSPC1’s ATPase activity (Lotz et al. 2003). Studies have demonstrated that Aha1 is essential in helping cells to overcome stressful conditions especially when HSPC1 levels are low, and Aha1 expression influences the activity of many oncogenic proteins including C-RAF, MEK1/2 and ERK1/2 (Holmes et al. 2008). Furthermore, treatment with 17-AAG results in sustained upregulation of Aha1 and silencing of Aha1 sensitises cells to 17-AAG treatment. NVP-AUY922 has also been shown to affect the levels of Aha1, and basal levels of Aha1 were found to be higher in NVP-AUY922-resistant non-small-cell lung cancer (NSCLC) cell lines than sensitive NSCLC cell lines (Garon et al. 2013). Future analysis of Aha1 may help to understand the mechanisms behind NVP-AUY922 resistance in these cells.

Clinical trial results had identified certain groups of patients who are more susceptible to HSPC1 inhibitions. A phase II trial in refractory metastatic colorectal cancer patients suggested that 53% of those who reported stable disease had KRAS-mutated tumour (Cercek et al. 2014). This is also supported by in vitro study showing that KRAS mutant tumours are more susceptible to HSPC1 inhibitions via degradation of STK33 (Azoitei et al. 2012). Two separate clinical trials investigating HSPC1 inhibitors in NSCLC patients have also identified those with ALK re-arrangement to be more susceptible to HSPC1 inhibitors (Sequist et al. 2010; Socinski et al. 2013). In contrast, trial investigating HSPC1 inhibitor in HER-2 (a sensitive client protein of HSPC1) positive breast cancer in 26 patients did not show any confirmed responses (Modi et al. 2013). The message thus far is consistently showing that HSPC1 inhibitors will likely be beneficial in a selected, susceptible group of patients. Moving forward, trials involving HSPC1 inhibitors will need to incorporate, either prospectively or retrospectively, steps to identify biomarkers that will identify susceptible patients.

#### Limitations

This study investigated the effects of three N-terminal HSPC1 inhibitors, which are 17-DMAG, NVP-HSP990 and NVP-AUY922, which for several years have been tested in a number of clinical trials on a variety of cancer types (Hong et al. 2013). Since the completion of this study, newer inhibitors such as resorcinol derivatives and C-terminal HSPC1inhibitors and also new strategies to target HSPC1 co-chaperones or client-protein interactions have begun to emerge as alternative routes to target HSPC1 activity (Shrestha et al. 2016). Nevertheless, this does not make the results from this study any less important, especially when considering HSPC1 inhibition in the clinical setting. The findings that the same cell type responded to the three N-terminal HSPC1 inhibitors differently is especially important in this context as it highlights that failure to achieve HSPC1 inhibition in a given patient using one inhibitor may be overcome by use of another similar drug. The results also highlight the importance of developing a model that can perhaps identify which type of inhibitor is most likely to work in an individual patient in the pre-clinical setting.

Another limitation is the use of a single cell line in this study. It could be argued that this may limit the strength of the findings. However, HSPC1 inhibition has been studied in other CRC cell lines, also with mixed results in terms of cell sensitivity and potentiation of other chemoagents (Tse et al. 2009; Powers et al. 2008; He et al. 2014). By using a single cell line to mimic a single patient, this study was able to demonstrate the different effects of inhibiting HSPC1 and the effect of combining these therapies with traditional chemotherapeutic agents. This direct comparison of multiple therapies in different combinations, using the same cell type, has not been performed before. The results show that given the ubiquitous and multifunctional nature of HSPs in humans, there may be many ways to target HSPs in a patient that will have similar desired effect. Moving forward, the challenge lies in finding the most suitable method for each patient.

## Conclusion

HSPC1 inhibitors are effective anti-cancer agents; however, their clinical applicability thus far is somewhat limited. This study showed even amongst N-terminal inhibitors that there are distinct differences in effects on CRC cells. In a clinical setting, this could mean that a single patient may show sensitivity to one inhibitor but resistance to another. Certainly, the results do indicate that dosing of NVP-AUY922 may need careful consideration to ensure sustained activity, and future clinical trials should put this into consideration. HSPC1 inhibitors also showed promise in potentiating current chemotherapeutic agents for CRC. Targeting HSPC1 is likely to be most effective in cancers where oncogenic drivers are sensitive client proteins. Therefore, prior to initiation of a clinical trial, it will be essential to identify a biomarker for sensitivity towards HSPC1 inhibitors.
